# The Controversial Link Between Human Papillomavirus Infection and Esophageal Health: An Exploratory Translational Study

**DOI:** 10.3390/pathogens15010096

**Published:** 2026-01-15

**Authors:** Maximilian Egg, Markus Wiesmüller, Bertram Aschenbrenner, Lili Kazemi-Shirazi, Werner Dolak, Behrang Mozayani, Reinhard Kirnbauer, Michael Trauner, Bettina Huber, Alessandra Handisurya

**Affiliations:** 1 Department of Dermatology, Medical University of Vienna, 1090 Vienna, Austria; maximilian.egg@meduniwien.ac.at (M.E.); markus.wiesmueller@meduniwien.ac.at (M.W.); bertram.aschenbrenner@meduniwien.ac.at (B.A.); reinhard.kirnbauer@meduniwien.ac.at (R.K.); bettina.huber@meduniwien.ac.at (B.H.); 2 Department of Internal Medicine III, Division of Gastroenterology and Hepatology, Medical University of Vienna, 1090 Vienna, Austria; lili.kazemi-shirazi@meduniwien.ac.at (L.K.-S.); werner.dolak@meduniwien.ac.at (W.D.); michael.trauner@meduniwien.ac.at (M.T.); 3 Department of Pathology, Medical University of Vienna, 1090 Vienna, Austria; behrang.mozayani@meduniwien.ac.at

**Keywords:** HPV6, esophageal squamous papilloma, esophageal papillomatosis

## Abstract

Evidence on the contribution of human papillomaviruses (HPVs) to the development of esophageal papillomas is still controversial. Esophageal papillomatosis (EP) is considered an exceedingly rare, but distinct entity within esophageal proliferations, with about 57 cases published so far. Tissues derived from an EP case and from non-EP esophageal papillomas were investigated for the presence of HPVs and virus-positive specimens were subsequently analyzed for transcriptional activity and surrogate markers of infection. Low-risk type HPV6 DNA was detected in a subset of the esophageal papillomatous tissues, including EP, and a variant isolate belonging to lineage A. In the EP tissue, the abundant expression of the viral E6/E7 mRNA and the presence of HPV6-specific E1^E4 transcripts, the latter indicative of productive viral infection, were detected. An analysis of HPV-specific neutralizing antibodies in sera obtained from the EP case during natural infection as well as after HPV vaccination revealed that, despite extensive manifestation, HPV6-specific antibodies were absent during natural infection and only elicited after repeated HPV immunizations. Although limited by a small sample size, this exploratory study suggests a possible involvement of HPV6 in the development of EP. Furthermore, this study may contribute to the evidence distinguishing EP from less extensive forms of non-EP esophageal squamous papillomas.

## 1. Introduction

Esophageal squamous papillomas are benign, mostly solitary tumors originating from the squamous non-keratinizing epithelium of the organ’s mucosa [1]. In rare occasions, multiple lesions or even extensive manifestation forms have been reported.

First described in 1977 [2], esophageal papillomatosis (EP) is characterized by the presence of numerous papillomatous proliferations throughout the esophagus which may progress to extensive, lawn-like growths or confluent masses. It was recently proposed to represent a distinct entity within esophageal papillomatous lesions [3,4].

The prevalence of EP is extremely low. Since its initial description up to 2023, a total of 53 cases have been reported in adults and children [4], with only three additional cases reported thereafter [5,6,7], alongside the EP case described here. Chronic mucosal irritation due to gastro-esophageal reflux disease, smoking, alcohol, caustic ingestion, or mechanical injury with subsequent regenerative proliferation have been implicated in EP development. Furthermore, genetic factors seem to have an etiological role, as indicated by the high coincidence of focal dermal hypoplasia, also known as Goltz syndrome, and EPs [4]. In addition, infection with human papillomaviruses (HPVs) have been implicated as responsible instigators.

HPVs’ early (E) genes E1 and E2 enable viral transcription and replication; the E4 gene product, expressed from the E1^E4 spliced mRNA, functions in cell cycle arrest and virus synthesis; and E5, E6, and E7 have transforming (oncogenic) characteristics and play a major role in tumorigenesis [8]. The late (L) genes L1 and L2 encode for the viral capsid proteins. The mucosal low-risk types HPV6 and -11 are commonly associated with the development of benign papillomas of the anogenital and oropharyngeal tract; persistent infection with high-risk types, predominantly HPV16 and -18, may cause cancers at these sites.

HPV’s role in the development of esophageal papillomatous lesions, however, is still controversial. Viral DNA detection rates of 30.9% were reported in a collective study comprising both esophageal papillomas and EPs, in 2013 [9]. In a recent review focusing solely on the 53 EP cases reported up to 2023 [4], the detection rate amounted to 37.9%, i.e., 11 out of 29 analyzed cases, with HPV6 and/or -11 found in 54.5% and HPV16 in 36.4% of the virus-positive cases. In addition, a comparison of the viral presence in cases of solitary esophageal papillomas as opposed to EPs revealed higher viral detection rates in EPs. Due to the scarceness of this entity and, hence, available evidence, the impact of HPV infection in the pathogenesis of EP is still unknown. Notably, EP is considered to harbor a substantial risk of malignant transformation, given that concurrent or subsequent esophageal squamous cell carcinomas (ESCCs) were reported in 22.6% of the EP cases [4].

As mucosal HPVs are implicated in having an impact on either papillomatosis or malignant transformation at anogenital and oropharyngeal mucosal sites, herein, we aimed to investigate whether HPVs may contribute to, at least a subset of, esophageal pathologies, in particular to EP.

## 2. Materials and Methods

### 2.1. Study Collection

In this exploratory translational study, patients who had presented to the Department of Internal Medicine III, Medical University of Vienna, between 11/2023 and 12/2024 with either EP or “non-EP” (herein defined as multiple esophageal squamous papillomas, but not regarded as papillomatosis) were included. This included a 54-year-old man with extensive EP and two patients (a 79-year-old woman and a 41-year-old man) with non-EP. The clinical details of the patients are described elsewhere (manuscript in submission).

### 2.2. DNA Isolation and PCR Analyses

Genomic DNA was isolated from archived formalin-fixed and paraffin-embedded (FFPE) EP and non-EP tissues employing the QIAamp DNA FFPE Tissue Kit (Qiagen, Hilden, Germany) according to the manufacturer’s instructions. The DNA was subjected to broad-spectrum PCRs using the established HPV-specific degenerate primers CP4/CP5 and PPF1/CP5 [10,11], both directed against the conserved papillomaviral E1 helicase region and capable of detecting a large spectrum of mucosal and cutaneous HPV types. Sanger’s sequencing was performed on the resulting amplicons on forward and reverse strands (Eurofins Genomics, Ebersberg, Germany) and the obtained sequences were blasted against the GenBank database to identify the HPV genotype.

Due to the wide genomic diversity of the HPV6 subtype, which had emerged as the sole HPV type identified in our esophageal tissues, nested PCRs were subsequently employed using specific primers, nHPV6L2, recognizing a conserved part within the HPV6 minor capsid protein L2. The amplicons were sequenced in both directions (Eurofins Genomics), which allowed us to discriminate between individual viral variants. The sequences were aligned to the HPV6b reference genome (GenBank accession number X00203) using the Unipro UGENE (v53.0) and the MUSCLE algorithm and the variant was identified by blasting the consensus sequence of the amplicon. All primer sequences are provided in Supplementary Table S1.

### 2.3. HPV E6/E7 Oncogene Analyses

The presence of the HPV6 oncogene E6/E7 mRNA was determined using RNAscope™ on 2–3 μm thick FFPE tissue sections derived from the virus-positive EP and non-EP specimens, following the manufacturer’s instructions. To verify tissue specificity, tissues derived from an anogenital wart, previously shown to be caused by HPV6, and normal, non-papillomatous, HPV-negative esophageal tissue were selected as appropriate positive and negative controls, respectively. Hybridization was performed, employing the RNAscope™ target probe specific to low-risk HPV6/11, as well as target detection, employing the Multiplex Fluorescent Reagent Kitv2 (both Advanced Cell Diagnostic Inc., Newark, CA, USA). The endogenous housekeeping gene POLR2A was used as the positive control to assess tissue RNA integrity and assay procedure, while the bacterial dapB gene probe was used as the negative control to assess background signals. For fluorescence staining, the Opal™ fluorophore Opal690 (diluted 1:900) was utilized, as well as 4′,6-diamidino-2-phenylindole (DAPI) for nuclear staining.

Images were captured (at 20× magnification) on a Vectra Polaris™ Automated Quantitative Pathology Imaging System (Akoya Biosciences Inc., Marlborough, MA, USA) and analyzed with the HALO^®^ image analysis platform (v3.6.4134.464, Indica Labs, Albuquerque, NM, USA). The quantification of HPV6 E6/E7 mRNA-positive cells was performed employing QuPath (v0.5.1, University of Edinburgh, UK). Total cell counts were determined based on DAPI nuclear staining and HPV6 E6/E7-positive cells were identified by the OPAL690 signal within previously annotated regions of interest in the epithelium. The proportion of positive cells (in %) was calculated for each sample. All samples were quantified using consistent settings and independently analyzed by two blinded assessors.

### 2.4. Determination of HPV Infectivity

The HPV’s E1^E4 spliced transcript is the most abundantly expressed gene product during the viral infection process and serves as a surrogate marker for active virus infection and disease severity [12]. To assess whether viral E1^E4 spliced transcripts were present in EP, RNA was first isolated from the EP tissues and reverse-transcribed into cDNA by standard methods. To verify specimen integrity, PCRs employing the endogenous control beta-actin were conducted. Samples were further subjected to nested PCRs using primers specific to the HPV6E1^E4 gene [13] in order to detect transcription. The primer sequences are given in the Supplementary Table S1. In addition, sequencing of the amplicons was performed on both strands to confirm the presence of correctly spliced viral E1^E4.

### 2.5. HPV-Specific Humoral Immune Responses During Natural Infection and After Vaccination

Pseudovirion-based neutralization assays were performed to assess the presence and levels of HPV-specific neutralizing antibodies in the HPV6b-positive EP patient during natural infection and after HPV vaccination with the commercially available nonvalent HPV vaccine (Gardasil™-9, Merck & Co., Inc., Rahway, NJ, USA). The vaccine contains non-infectious, adjuvanted protein antigens from nine different HPV types, specifically the low-risk HPVs 6/11 and the high-risk HPVs 16/18/31/33/45/52/58. Pseudovirions (PsVs), designed to encapsidate a reporter plasmid encoding for secreted alkaline phosphatase (SEAP), were generated for the HPV genotypes 6 and 16 and purified via Optiprep gradient centrifugation, according to standard methods [14]. For the neutralization assays, the PsV preparations from either HPV6 or HPV16 were pre-incubated with patients’ sera obtained at indicated time points in four-fold serial dilutions prior to the in vitro infection of 293TT cells. The levels of SEAP released from infected cells into the culture supernatant inversely correlate with the inhibition of PsV infection and, thus, can be used to determine the neutralizing capacities of the sera [15,16]. The SEAP signals were measured three days after infection in a microplate reader (Opsys MR, Dynex Technologies, Aspect Scientific Ltd., Tarporley, UK). Serum dilutions showing at least a 50% reduction in SEAP activity compared with a control, HPV-negative serum at the same dilutions that were considered neutralizing [16].

## 3. Results

### 3.1. Detection of an HPV6 Variant

**Figure 1:**
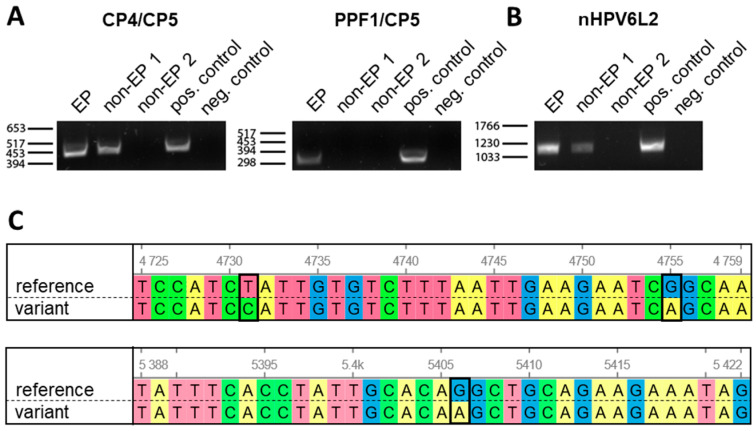
Detection and identification of HPV in the esophageal tissues. (**A**) Broad-spectrum HPV PCRs with primer pairs CP4/CP5 and PPF1/CP5 were used for detection of HPVs in the esophageal tissues. Tissues were obtained from a patient with extensive papillomatosis (EP) and from two patients with multiple esophageal squamous papillomas (non-EP 1 and non-EP 2). Amplicons at the expected base pair sizes were observed in the EP patient and in the non-EP patient 1 ((**left**); CP4/CP5 at 450 base pairs) and in the EP patient ((**right**); PPF1/CP5 at 280 base pairs), but not in both non-EP patients. (**B**) To determine the individual HPV6 variant, a nested PCR using nHPV6L2 primers, recognizing a conserved part within L2, was performed. HPV6L2-specific bands at the expected size of 1073 base pairs were observed in the virus-positive tissues, including the sample derived from the EP patient. DNA isolated from an HPV6-positive anogenital wart (pos. control) and water (neg. control) served as the respective positive and no-template controls in the PCRs. (**C**) Sequencing of the HPV6L2 isolates derived from the esophageal tissues as compared to the HPV6b reference genome (GenBank accession number X00203) revealed a virus variant belonging to the HPV6 lineage A. The three single nucleotide polymorphisms detected in both virus-positive samples are annotated.

In two out of the three patients with esophageal pathologies, broad-spectrum HPV PCR analysis yielded positive signals at the expected base pair size (Figure 1A). Amplicons were observed with both CP4/CP5 and PPF1/CP5 primer pairs in the tissue obtained from the EP patient. One non-EP patient was positive for CP4/CP5, while the other non-EP patient was consistently negative. Surprisingly, the low-risk mucosal HPV6 genotype was identified by sequencing in all amplicons obtained from the virus-positive esophageal tissues, hence, investigations subsequently focused on this particular genotype.

Given the reported genomic diversity of HPV6, the DNA of the virus-positive patients was next subjected to PCR using primers directed against the HPV6L2 protein, synthesizing an amplicon that was described adequate to differentiate between individual HPV6 variants (Figure 1B). Blasting of the amplicons’ sequences identified the same viral variant in both samples. The amplicons of the identified isolate showed 100% homology in the nucleotide sequence at positions 136–1176, when the A in the L2 start codon had been set at position 1. In addition, three single nucleotide polymorphisms, specifically T > C at position 4731, G > A at position 4755, and G > A at position 5406, were detected, consistent with a specific variant previously deposited in GenBank as isolate CAC251c (accession number FR751321) and as isolate 50 (accession number HG793858) (Figure 1C) [17,18]. This variant belongs to the HPV6 lineage A, which is closely related to the former HPV6b prototype [19].

### 3.2. Detection of HPV6 E6/E7 Oncogene mRNA

**Figure 2:**
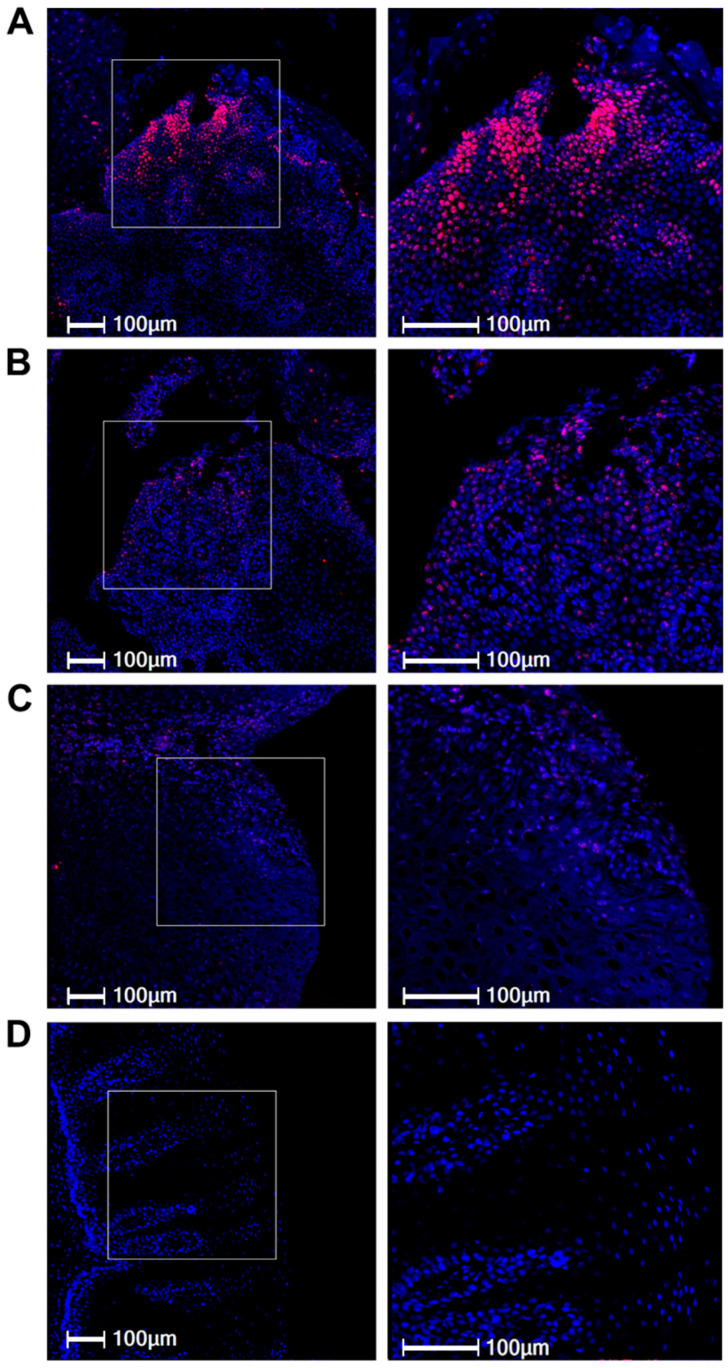
Detection of HPV6 oncogene E6/E7 mRNA using RNAscope in esophageal tissues. (**A**) Tissue samples obtained from the papillomatous area of the patient with extensive papillomatosis (EP) contained substantial HPV6 E6/E7. Strong positive signals (red) were detected in numerous foci throughout the epithelium, with pronounced accumulation in the suprabasal layers. (**B**) The adjacent, non-papillomatous area in the same EP patient showed fewer positive signals for HPV6 E6/E7. (**C**) In contrast, papillomatous tissue from the HPV6b-positive, non-EP patient exhibited only a few scattered cells with weak HPV6 E6/E7 mRNA positivity. (**D**) Normal, healthy esophageal tissue lacked HPV6 E6/E7 mRNA expression. Left panel: low-power overview (10× magnification). Right panel: higher-power view (20× magnification) of the area indicated in the inset. Nuclei were counterstained with DAPI (blue). The scale bars on the images represent 100 mM.

In the esophageal tissues obtained from the two HPV6b-positive patients, variable amounts of the papillomaviral E6/E7 mRNA were detected. In the EP patient, samples taken from the papillomatous area contained high amounts of viral oncogene mRNA (Figure 2A). The positive signals were detected in numerous foci throughout the papillomatous epithelium. Similarly to other HPV-infected tissues, the viral oncogenes were primarily expressed in the suprabasal layers of the non-keratinizing epithelium, showing a pronounced accumulation in the most superficial layers. In the adjacent, non-papillomatous area of the same EP patient, the viral E6/E7 mRNA was detectable in a similar pattern, albeit at much lower levels (Figure 2B). In contrast, in the non-EP HPV6b-positive patient, scattered and only weakly HPV6 E6/E7 mRNA-positive cells were detected in the suprabasal layers (Figure 2C). Viral E6/E7 mRNA was absent in normal esophageal mucosa (Figure 2D), thus, corroborating the specificity of the results. The positive control, an HPV6-positive anogenital wart consistently showed positive signals for HPV6 E6/E7 (Supplementary Figure S1).

The quantitative comparison of signals revealed that the percentage of HPV6 E6/E7-positive cells was higher in the papillomatous area than in the adjacent regions (4.18% versus 1.61%, respectively) of the EP patient. In addition, positive signals in the papillomatous areas were 7.35-fold higher in the EP patient compared with the papilloma derived from the non-EP patient (0.57%). However, the values have to be regarded with caution due to the small sample size.

### 3.3. Viral Transcriptional Activity in EP

Given the presence of viral E6/E7 mRNA in the EP patient, the EP tissue was further examined for markers indicative of productive papillomaviral infection. The HPVs E1^E4 spliced product is the most abundantly expressed viral mRNA during infection and, hence, serves as the surrogate marker [12].

**Figure 3:**
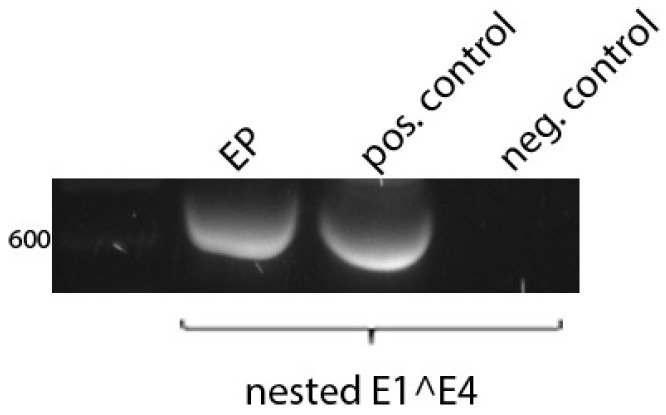
Detection of HPV6 E1^E4 spliced transcripts in tissues obtained from extensive papillomatosis (EP) via nested PCR. Specific transcripts were qualitatively detected in the EP tissue at the expected 653 base pair size. The positive control was derived from an HPV6-positive anogenital condyloma (pos. control). Water served as the no-template control (neg. control).

Using nested PCR, HPV6 E1^E4-specific transcripts were qualitatively detected in the EP tissues (Figure 3). To verify the specificity of the obtained transcripts, the bands migrating at the expected base pair size were additionally purified from agarose gel and sequenced to determine the E1^E4 splicing sites in forward and reverse. The sequencing results were confirmative and, thus, the presence of the HPV6-specific E1^E4 transcripts indicated a productive HPV6 infection within the EP tissues.

### 3.4. HPV-Specific Humoral Immune Responses

**Figure 4:**
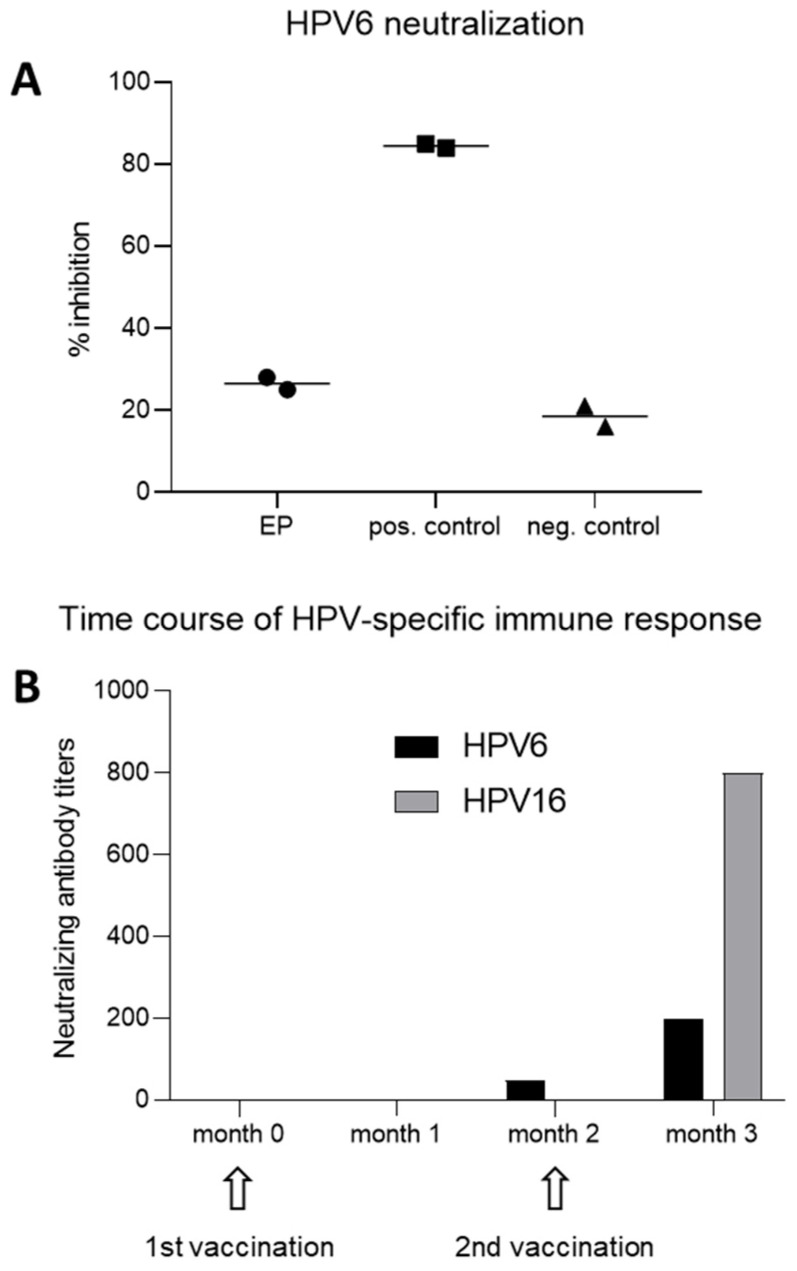
HPV type-specific humoral immunity in the patient with EP during natural infection and after HPV vaccination. (**A**) Sera obtained during natural infection, i.e., prior to HPV vaccination, showed neutralization of HPV6-derived pseudovirion infection at levels similar to the HPV-negative control serum (neg. control). In contrast, the control serum obtained from an immunocompetent, HPV-vaccinated individual (pos. control) contained neutralizing antibodies capable of inhibiting HPV6 pseudovirion infection. (**B**) Time course analysis of serum samples obtained from the EP patient revealed lack of detectable HPV6-specific titers at month 0 (prior to vaccination) and month 1 (one month after the first immunization). Minimal antibody titers were detected at month 2 (two months after the first immunization and the time of the second HPV vaccination), that increased at month 3 (one month after the second immunization). HPV16 neutralization was detectable only at month 3. The timepoints of HPV vaccination of the patient are indicated.

To investigate whether a transcriptionally active and productive esophageal HPV infection induces an efficient humoral immune response, the sera derived from the patient with HPV6b-induced EP were investigated for the presence of neutralizing HPV6-specific antibodies. Despite the pronounced clinical presentation, neutralizing antibodies were not detected in the EP patient, indicating a lack of virus-specific humoral immunity during natural infection, as the inhibition of HPV6 PsV infection by the patient’s serum (diluted at 1:80) was demonstrated at levels of 26.5% (±2.1%), which are relatively similar to the levels obtained with the negative control at 18.5% (±3.5%) (Figure 4A). In contrast, the positive control serum, known to contain high levels of neutralizing HPV6-specific antibodies, was able to inhibit HPV6 PsV infection at levels of 84.5% (±0.7%).

To possibly prevent the further spread of the virus to adjacent non-infected tissues and to prevent potential recurrence after the endoscopic mucosal resection of the lesions, the patient received two injections (months 0 and 2) with the nonavalent HPV vaccine, off-label. The third vaccination was scheduled to be given within a year after initiation, but the patient was lost to follow-up. Analyses of pre- and post-vaccination serum samples for neutralizing activity (Figure 4B) were performed for HPV6 as the representative type for low-risk HPVs and the causative type, and for HPV16, the representative for the group of high-risk HPVs, both included in the vaccine formulation. Surprisingly, HPV6 antibodies were undetectable at month 1 and hardly detectable (at a titer of 50, given that a cut-off value of <50 was considered negative according to the negative control sera) at month 2, i.e., after one immunization but prior to the second immunization. After the second vaccination at month 2 had been performed, neutralizing antibodies against HPV6 were mounted, albeit at low titers of 200, as shown at the 3-month time point. This moderate humoral response was not only confined to the HPV6 genotype, as antibody titers against HPV16 were only induced after the second vaccination. However, titers against HPV16 were four-fold higher, at 800, than the titers observed against HPV6.

## 4. Discussion

Cases with EP have increasingly been reported in the past two decades, although the total number of reports are very low, with about 57 cases published since 1977 [4,5,6,7]. While EP has been regarded for a long time as an extensive manifestation form of esophageal papillomas, certain inherent characteristics indicate that EP represents a distinct entity [3,4].

Herein, an HPV6 variant belonging to lineage A was detected in a subset of esophageal proliferations. This lineage encompasses the former prototype HPV6b and its closely related variants and has been detected worldwide, with a predominance in Asia [20]. This particular variant has also been detected in anogenital warts derived from two patients in Slovenia [17,18], but, to the best of our knowledge, has not yet been reported in esophageal tissues. While the intratype’s molecular diversity appears to be of significance, the exact clinical implications have not yet been fully unraveled and knowledge whether a certain HPV6 variant confers enhanced infectivity, immune evasion, or vaccine resistance is still lacking [18,21].

Low-risk HPVs are commonly associated with the development of benign oral, laryngeal, pharyngeal, and anogenital papillomas. Their role, however, in the pathogenesis of esophageal papillomatous proliferations is still controversial and data are scant. In our EP patient, the presence of the HPV6 E6/E7 mRNA and E1^E4 transcripts supports the notion of non-negligible viral activity in the esophageal mucosa and the coordinated activity of the virus’ early genes, which, in turn, may contribute to papilloma growth and/or the persistence of lesions [22]. Even though the exact mechanism has not yet been fully unraveled at this anatomical site, here, several pathways may be involved. For instance, the viral E6-mediated modulation of p53-dependent pathways, albeit without inducing efficient p53 degradation, and the induction of the cell cycle re-entry of differentiating keratinocytes via interactions of E7 with members of the pRb family could play a role [23]. In addition, the low-risk E7 is capable of inducing the degradation of p130, which could contribute to epithelial hyperplasia, a hallmark of papillomatosis [24]. The viral E1^E4 could disrupt the keratin network and support viral genome amplification and virion release during epithelial differentiation [12] to subsequently promote viral spread and/or contribute to the extensive manifestation form of the EP. It is furthermore conceivable that in our EP patient, or even in EP in general, HPVs may take advantage of the esophagus’ microenvironment with its unique stratified squamous lining and diverse microbiome via hitherto unknown mechanisms and/or may be favored by other local chronic predisposing factors, such as repeated irritation by acid reflux or solid food particles, low pH or oxygen levels, and chronic inflammation, all of which affect mucosal integrity, to facilitate papilloma growth or maintenance.

The detection of HPV DNA, however, does not necessarily implicate that infection of the cells has actually occurred, as the presence of the virus may merely represent a viral deposition or the contamination of the tissue’s surfaces. In this regard, previous reports have shown that the virus load of skin tumors can be reduced by the removal of the outermost skin layers [25]. However, this may not be the case in our samples, as the viral E6/E7 oncogene was demonstrated throughout the epithelium in our EP tissues. This is in contrast to a recent large study conducted by Li et al. encompassing around 140 patients with esophageal papillomas from the US and China [26], which failed to show the presence of papillomaviral E6/E7 mRNA employing the same technique. The causes for the discrepancy have not been deciphered but may reflect technical differences or differences in the study populations. For instance, Li et al. used hybridization probes which allow the detection of E6/E7 mRNA of a total of 10 low-risk HPV or 18 high-risk subtypes, but while these probe cocktails target a larger range of genotypes, they assumedly might differ in the sensitivity to HPV6. Furthermore, the samples from Li et al. had mostly been derived from single and only few from multiple papillomas, but not from patients with EP. In contrast, our sample containing abundant E6/E7 mRNA had been obtained from EP, which may explain the observed differences. In line with this assumption, the sample which had derived from the non-EP form, consisting of a large papilloma and several smaller satellite lesions, had only a few scattered cells that were weakly positive. Together with the previous analyses on the HPV status in which EP cases had higher viral detection rates as opposed to patients with solitary and few multiple papillomas (manuscript in submission), this further indicates a differential expression between “normal”, i.e., solitary or few multiple, papillomas, and EP. Nevertheless, further studies are required to distinguish EP as a separate entity from esophageal papillomas in general, as herein only material from one EP patient was available.

Interestingly, despite the large affected surface within EP from which the virus can assumedly be easily shed in substantial quantities, and despite repeated irritations by solid foods and endoscopic procedures which may enhance auto-inoculation and/or re-infection, HPV-specific humoral immune responses to the virion capsid were not elicited during natural infection. This is in line with previous observations in the anogenital tract, where the presence of large, extensive warts does not automatically induce a humoral immune response. The HPVs infect basal epithelial cells without causing cell lysis, concomitant inflammation, or viremia and the L1/L2 are expressed only late in the upper epithelium, thus minimizing systemic antigen exposure [27]. Together with the inherent immuno-evasive properties of the HPVs, mediated, for instance, by the downregulation of type I interferons and the inhibition of MHC class I and II expression by the viral early proteins [28], this further limits immune recognition and may hamper or delay neutralizing antibody responses. The lack of a protective humoral immune response, in turn, may facilitate the spread of the virus and could have allowed for the extensive manifestation, at least in our EP patient.

Although HPV vaccination does not exert therapeutic effects, vaccination-induced type-specific protection against the infection of non-infected tissues with subsequent reductions in the spread and/or recurrence of anogenital and oropharyngeal diseases have been reported, including in several studies and meta-analyses [29,30,31]. Furthermore, for recurrent respiratory papillomatosis, HPV vaccination administered as an adjuvant was shown to increase the interval between surgeries and to reduce the number of surgical procedures required [32]. However, discordant results were reported in other studies and meta-analyses [33,34,35]. Given the unresolved issue even in the more established HPV-related diseases, the impact of vaccination on progression or recurrence in the esophagus has not yet been addressed.

In our EP case, HPV immunizations induced antibody responses detectable for both HPV6 and HPV16, albeit antibody titers were unexpectedly low and delayed. Surprisingly, neutralizing antibody titers against HPV6 remained low, even after repeat immunization. In recurrent respiratory papillomatosis patients, HPV-specific serum antibodies were elicited during natural infection [36,37], indicating that at this closely located anatomical site, viral recognition takes place and site-specific immune tolerance due to the polarization of the adaptive immune response to a T helper type 2-like or T regulatory phenotype occurs. This may subsequently allow for the inhibition of viral clearance and disease persistence [38,39,40]. Whether this specific inadequacy is also applicable in EP is unknown. Notably, in our patient, concomitant cellular and humoral immunodeficiencies were absent and previous vaccinations against, e.g., Hepatitis A/B and Measles–Mumps–Rubella had generated good immune responses. In this regard, once the role of HPVs is unequivocally established in EP, the possible preventive role of HPV vaccination, either prior to viral infection or adjunct to surgical treatment, in patients with EP or esophageal proliferations would be interesting to be addressed in future.

So far endoscopic and conventional surgical management are the mainstay of therapy [3]. Traditional medicine agents have increasingly been explored for their antiviral and anti-proliferative effects in HPV-associated epithelial disorders [41]; however, evidence for their therapeutic efficacy in EP is currently insufficient.

In conclusion, although these findings are exploratory and should be interpreted with caution given the limited number of cases herein, this study suggests the potential involvement of low-risk HPV6 in the development of a subset of esophageal pathologies, in particular EP. Further studies are needed and until proven otherwise, the monitoring of affected patients is warranted. It would be of interest to investigate a larger number of EP cases, which was unfortunately hampered herein due to the scarcity of this pathology. Nevertheless, this study may contribute to the available evidence that EP is an entity distinct to esophageal squamous papillomas.

## Data Availability

The data that support the findings of this study are available from the corresponding author upon reasonable request.

## Supplementary Materials

The following supporting information can be downloaded at: https://www.mdpi.com/article/10.3390/pathogens15010096/s1, Figure S1: RNAscope analysis; Table S1: List of PCR primers.

## Abbreviations

The following abbreviations are used in this manuscript:

DAPI	4′,6-diamidino-2-phenylindole
E	early
EP	esophageal papillomatosis
ESCC	esophageal squamous cell carcinoma
FFPE	formalin-fixed and paraffin-embedded
HPV	human papillomavirus
L	late
PsV	pseudovirion
SEAP	secreted alkaline phosphatase
