# Supplementary material for: The Controversial Link Between Human Papillomavirus Infection and Esophageal Health: An Exploratory Translational Study
Source: Pathogens. 2026 Jan 15;15(1):96. doi: 10.3390/pathogens15010096 (PMC12845082; doi:10.3390/pathogens15010096)
Supplement: Supplementary file 1 [file pathogens-15-00096-s001.zip › pathogens-4080407-supplementary.pdf]

Supplementary Table S1. List of PCR primers

Primer		sequence
CP4	forward	5′ - ATG-GTA-CAR-TGG-GCA-TWT-GA - 3′
CP5	reverse	5′ - GAG-GYT-GCA-ACC-AAA-AMT-GRC-T - 3′
PPF1	forward	5′- AAC-AAT-GTG-TAG-ACA-TTA-TAA-ACG-AGC - 3′
(n)HPV6 L2	outer forward	5′ - TGG-CAC-ATA-GTA-GGG - 3′
	outer reverse	5′ - CTA-AAG-GGT-GTT-CCC - 3′
	nested forward	5′ - GAT-GTA-ATT-CCT-AAG-GTG-GAG - 3′
	nested reverse	5′ - AAC-AGG-TCA-TTA-GGA-AGT-GA - 3′
HPV6E1^E4	outer forward	5′ - TGC-AAC-AGC-TTC-TGT-TGG-GAA-CAC - 3′
	outer reverse	5′- CGG-GAC-AGT-AAC-ACA-CAA-GTA-GAG - 3′
	nested forward	5′-ACA-TAG-TGT-GTC-CCA-TCT-GCG - 3′
	nested reverse	5′-GTT-CCT-GCA-GCT-ATT-TGT-ACA-GG - 3′

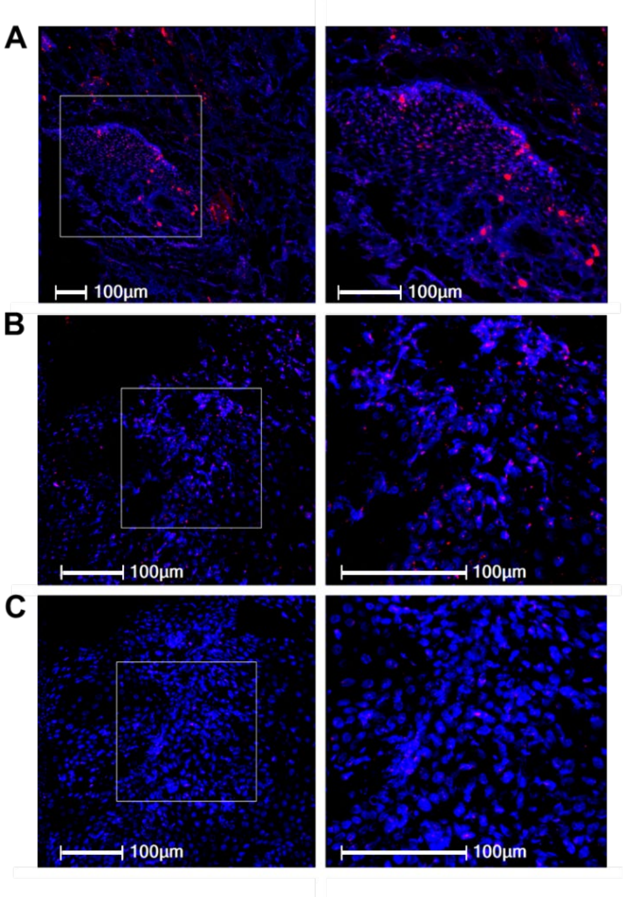

Supplementary Figure S1. RNAscope analysis. (**A**) HPV6 E6/E7 mRNA (red) in a HPV6-positive anogenital wart, which served as the positive control. (**B**) Positive signals to the endogenous housekeeping gene POLR2A (red) show the integrity of the RNA in the esophageal papillomatosis tissues. (**C**) The bacterial dapB gene probe was employed as negative control to assess background signals in the esophageal tissues. The depicted scale bars represent 100 μM.
